# A Framework for the Estimation of Damping Ratio of Glued–Laminated Buildings by Use of Analysis in the Time Domain

**DOI:** 10.3390/ma18071545

**Published:** 2025-03-28

**Authors:** Saule Tulebekova, Haris Stamatopoulos, Kjell A. Malo

**Affiliations:** Department of Structural Engineering, Norwegian University of Science and Technology, NO-7491 Trondheim, Norway; kjell.malo@ntnu.no

**Keywords:** timber, damping, modeling, tall timber building

## Abstract

The increased interest in tall timber buildings has led to the need for more accurate prediction models. With inherently low mass and stiffness properties, multi-story buildings made of timber are susceptible to wind-induced vibrations, which can result in discomfort for the occupants. Multiple experimental and numerical studies investigating natural frequencies and mode shapes of timber buildings can be found in the literature. However, modeling the damping properties in timber buildings has not been studied fully yet. This study presents a framework for the estimation of the global damping ratio of glue–laminated-frame buildings by use of linear-elastic finite element modeling. Using stiffness-dependent Rayleigh damping and the dynamic analysis in the time domain, it was demonstrated that the predictions of the FE model for the damping ratio were within the range of the results obtained by on-site measurements. The case study of the tallest all-timber building in the world (Mjøstårnet, Norway) was used to demonstrate the framework using extensive small- and large-scale experimental data. The parametric study identified the damping ratio in the diagonals and material damping ratio in the glue–laminated timber as the key parameters influencing the damping ratio of the whole building.

## 1. Introduction

Wind-induced accelerations within acceptable limits at the serviceability limit state (abbr. SLS) are usually the governing design criterion for multi-story timber buildings, at least in non-seismic-prone areas. Wind-induced accelerations are highly dependent on the dynamic properties: eigenfrequencies and the damping ratios of the buildings. Numerous in situ vibration tests on multi-story timber buildings have been conducted recently (e.g., [1,2,3,4,5]). These studies contributed significantly to the experimental database of multi-story timber buildings, which is essential for understanding their wind-induced behavior. Along with that, multiple numerical studies have been conducted to understand the dynamic behavior of timber buildings through investigation of the modal properties (e.g., [6,7,8,9,10]). Recent theoretical studies on the response of moment-resisting timber frames under service-level wind loading [11] have demonstrated the significant influence of the damping ratio on the acceleration, both by use of time-history, linear-elastic analysis and by applying the approximate method of EN1991-1-4 [12]. Similar theoretical findings have been obtained for post-and-beam timber structures [11].

The investigation of the environmental variability of the dynamic properties of timber buildings showed that while the natural frequencies exhibited small seasonal variations, no damping variation related to the environmental conditions was observed [13,14,15].

Among the vibration properties, damping is an important parameter related to the energy dissipation within the building that significantly impacts the dynamic response of the building. The general definition of damping is the process of steadily diminishing the amplitude of vibrations. Damping is a complex process in timber building systems, encompassing energy dissipation properties within timber material and at the structural level due to dynamic loading. Contributing factors to damping in the building include material damping (energy dissipation within the material), friction and interaction between the structural members and connections, and aerodynamic damping due to interaction between the airflow and the building exterior.

At the material level, damping in timber has been studied through experimental investigations by Labonnote et al. [16]. In this study, Labonnote conducted an extensive experimental campaign on glue–laminated timber (glulam) and solid wood beams. The campaign consisted of a series of experimental modal analysis tests by use of a modal hammer and accelerometers to determine the eigenfrequencies, eigenmodes, and damping ratios. According to the findings of the study, the damping ratio of slender beams, where bending deformation is dominant, is approximately 0.5%, while for more stocky beams, where shear deformation is more significant, the damping ratio is approximately 1.0%.

A recent study on the dynamic response of timber composite floors [17] has demonstrated that stiffness-dependent Rayleigh damping was more suitable to represent energy dissipation of timber than Rayleigh damping dependent on both mass and stiffness. This conclusion was reached by attempting to model the steady-state acceleration of a floor subjected to forced, resonant vibrations.

On a sub-assembly level, the framework for the estimation of damping has been applied and experimentally verified by Vilguts et al. [18] by use of FE analysis and tests on a mock-up timber structure with glulam elements and moment-resisting connections. The estimation of the damping ratio from the FE model of the mock-up was obtained by use of the stiffness-dependent Rayleigh damping model and the dynamic analysis in the time domain. The damping ratio obtained from the model was similar to the experimental results, demonstrating the suitability of the modeling approach.

According to EN1991-1-4, the viscous damping ratio for timber bridges shall be within the range of 1.0–1.9% [12]. Since the Eurocodes do not provide damping ratio values for timber buildings, engineers commonly use the damping ratios for bridges in their timber building design.

The framework for the estimation of the global damping ratio in glulam-frame buildings has not yet been studied in the existing literature. To this end, the present study presents a framework for estimating the damping ratio of a glulam building by use of the dynamic analysis of free vibrations in the time domain. The present study uses stiffness-dependent Rayleigh damping to simulate energy dissipation. This approach is chosen due to its simplicity but also due to limited evidence from the previous studies both on sub-assembly and large-scale structural levels as presented in the studies by Bjerve et al. [17] and Vilguts et al. [18].

## 2. Proposed Framework

**Figure 1:**
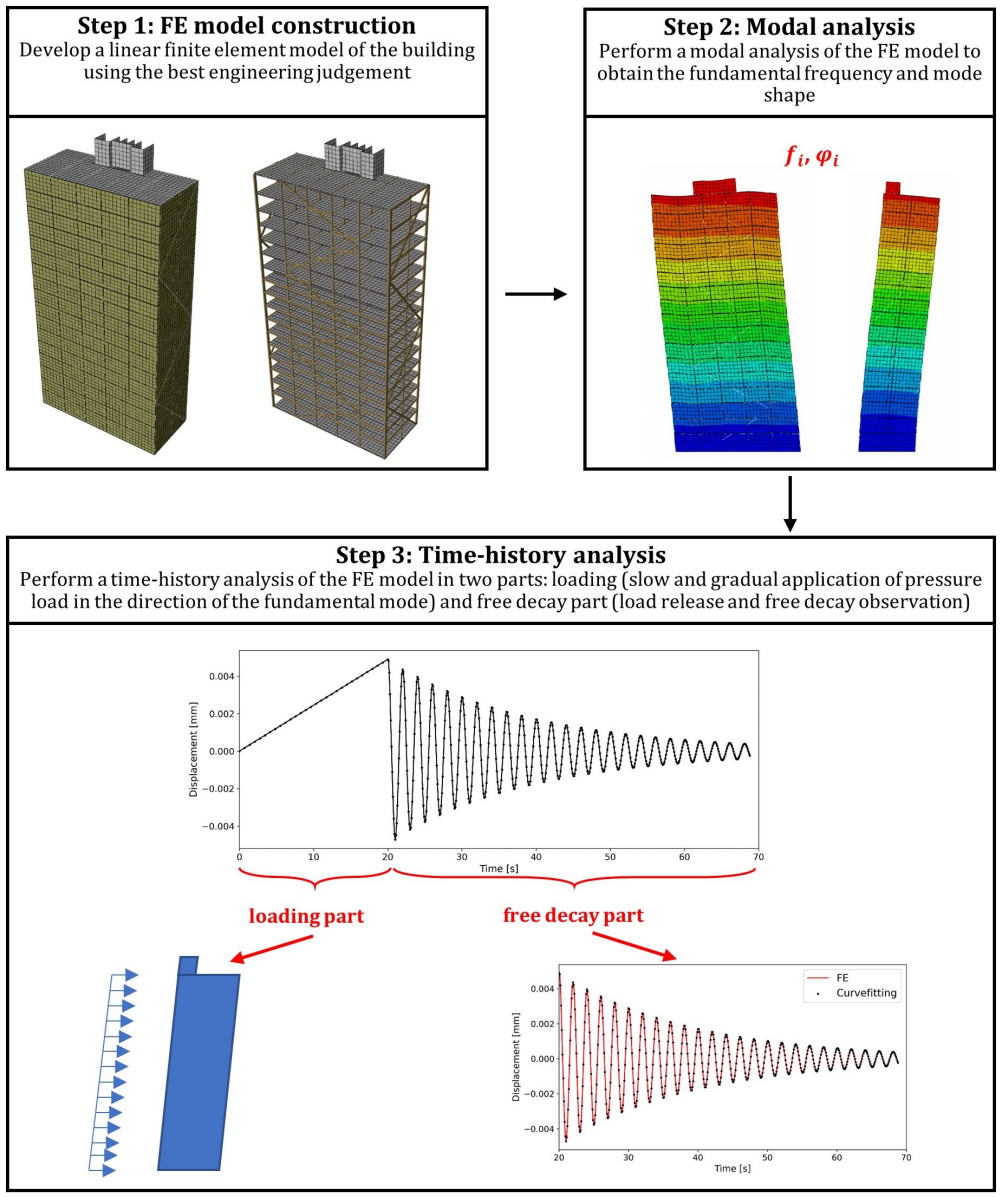
Proposed framework for modeling and analysis.

As mentioned earlier, there is a lack of finite element models aiming at the estimation of the global damping ratio in timber buildings. This section proposes the methodology for estimating the global damping ratio from the linear-elastic model of the timber building using stiffness-dependent Rayleigh damping. The framework for estimating damping in multi-story glulam buildings consists of three main steps: developing a detailed numerical model, conducting modal analysis to obtain the natural frequencies and mode shapes, and conducting the dynamic analysis of free vibrations in the time domain to estimate the global damping. The steps presented in Figure 1 are described below:

- Step 1: The framework depends on developing a linear-elastic finite element model. The numerical model should accurately represent the building’s actual design, incorporating the accurate geometry, structural components, and material properties. Stiffness and damping properties shall be assigned to the main timber elements and glulam connections.
- Step 2: Once an accurate model has been developed, modal analysis is conducted to obtain the structure’s undamped natural frequencies and mode shapes.
- Step 3: The last step is to estimate the structure’s global damping ratio from dynamic analysis in the time domain. The process is carried out in three stages. In the first stage, uniform loading is gradually applied on one face of the structure. Then, in the second stage, the load is released, and the structure is allowed to vibrate freely. Finally, the damping ratio and fundamental frequency of the structure are calculated from free vibrations.

The damping ratio for timber buildings is essential for estimating the wind-induced accelerations related to the comfort criteria. The purpose of this framework is to provide a straightforward method for estimating the damping ratio of timber buildings using a common finite element software with linear properties and a generalized damping approach. The parametric study is conducted to identify the key parameters influencing the damping ratio of the building. The proposed framework can be applied to a building with regular geometry, i.e., buildings with vibration response dominated by the fundamental mode with insignificant contributions from the higher-order modes.

## 3. Case Study

This section describes the case study building, Mjøstårnet [19], which was selected to apply and evaluate the proposed framework. The choice of the case study building was based on the availability of the laboratory and field measurements. The stiffness and damping properties of the Mjøstårnet-type dowelled connections were studied by Frette et al. [20]. Moreover, extensive field tests were conducted to determine the dynamic properties of the building, such as natural frequencies, mode shapes, and damping ratios [5]. The data from the laboratory and field experiments allows for the demonstration of the practical application of the framework. In this section, a building description is given first, followed by the results from the laboratory and field experiments. It is important to note that the presented laboratory and field studies have already been published, and the key descriptions and the summary of the results are given in this section for completeness.

### 3.1. Building Description

**Figure 2:**
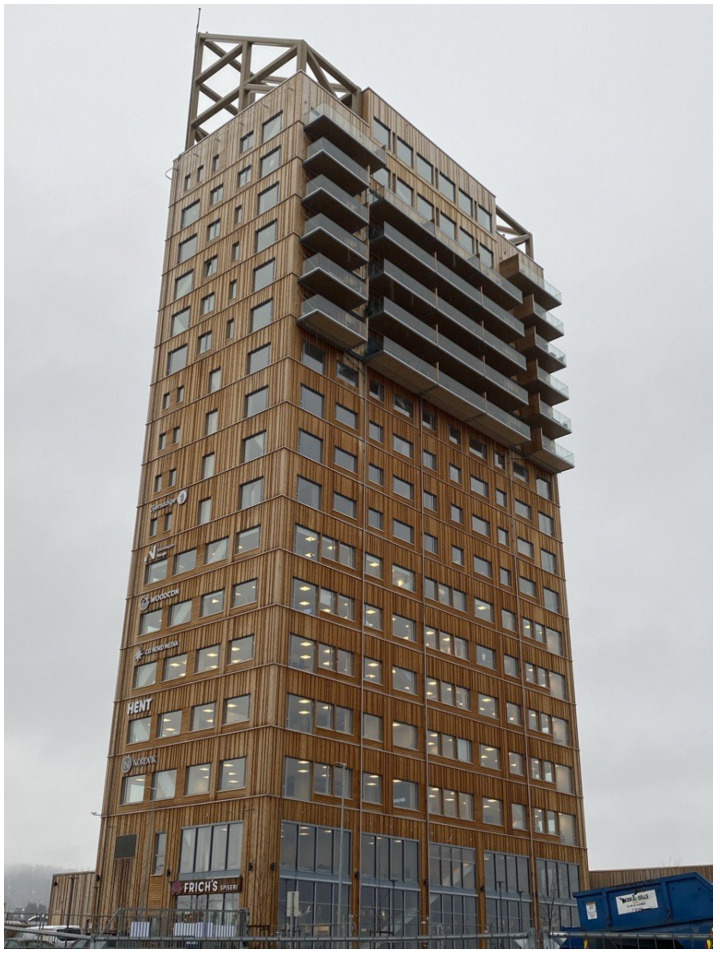
Case study building Mjøstårnet.

This section describes the building used as a case study for the proposed framework evaluation. The building is Mjøstårnet (the Mjøsa Tower, Figure 2 [19]) and with a total structural height of 85.4 m, it is the tallest all-timber building in the world as of November 2024 to the authors’ best knowledge [21]. The building has a rectangular shape with dimensions of 36.3 m by 15.7 m. The main lateral load-resisting system consists of the glulam truss. The shafts are made of cross-laminated timber (CLT) panels and are not designed as part of the lateral load-resisting system. The bottom 10 floors are composite timber decks, and the top 8 floors are reinforced concrete decks. The facade of the building is made of thin timber cladding and internal partitions are made of plasterboards.

### 3.2. Laboratory Tests of Connections

Large- and small-scale laboratory experiments on the stiffness and energy dissipation of dowelled connections in glulam elements have been conducted previously by Frette et al. [20]. The experiments and the detailed data have been analyzed and published previously [20,22], and a brief description of the experiments and the key relevant results are presented in this section. The stiffness and the energy dissipation of connections are input parameters to the finite element (FE) study presented in this paper. Connections with multiple slotted-in steel plates and dowels are used in Mjøstårnet to connect the diagonal elements to the columns and the beams to the columns. According to EN1995-1-1 [23], the stiffness per shear plane per dowel of connections with slotted-in steel plates can be obtained by Equation (1). This equation also provides the value for dowels with diameter *d* = 12 mm and glulam of strength class GL30c (according to EN14080 [24], the mean density of GL30c is $\rho_{\mathrm{mean}}=430\;{\mathrm{kg}/m}^{3}$) which corresponded to the connections of Mjøstårnet.

(1) $$K_{ser}=\frac{2\cdot \rho_{\mathrm{mean}}^{1.5}\cdot d}{23}=9.3\;\mathrm{kN}/\mathrm{mm}$$

As indicated by Equation (1), the connection stiffness is proportional to the number of dowels, and thus, no group effects are accounted for. Moreover, Equation (1) is independent of the angle between the force and the grain direction.

**Figure 3:**
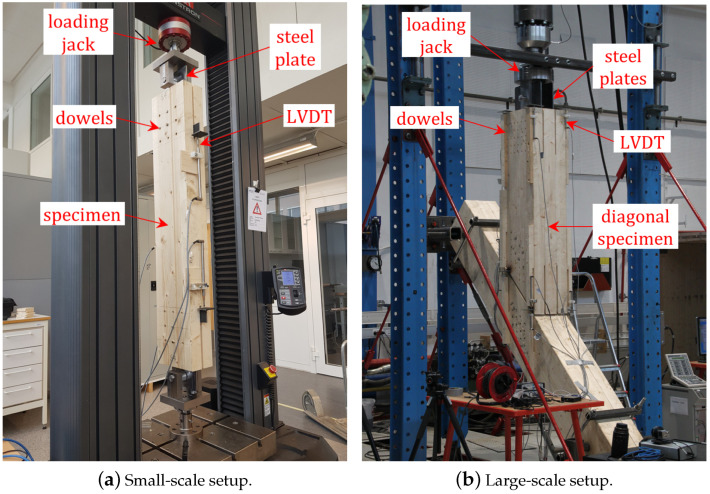
Small- and large-scale laboratory experiments (photos by Frette et al. [20]).

Frette et al. [20] carried out a series of non-destructive tests on such connections subjected to cyclic, service-level loading. The test setups included small- and large-scale tests (Figure 3). The small-scale tests are shown in Figure 3a and included the specimen with dimensions of 220×115 mm^2^ and length of 1200 mm, which was cut from the inner cross-section of GL30c consisting of laminations of strength class T15 according to EN338 [25]. The large-scale tests are shown in Figure 3b and included two blocked glulam elements, each with a cross-section of 450×345 mm^2^ connected and the loading jack with two connections made of steel plates. In both tests, the materials were similar to the connections of Mjøstårnet (12 mm-dowels and GL30c glulam).

The number of dowels was varied in these tests. At first, each specimen was tested non-destructively with a small number of dowels, and gradually the number of dowels increased. A clear group effect was observed with respect to the stiffness per shear plane per dowel. For a small number of dowels, the experimentally determined stiffness values per shear plane per dowel were, in general, similar or higher compared to the prediction by Equation (1). However, as the number of dowels increased, the stiffness values per shear plane per dowel converged to a significantly lower value. The connections of Mjøstårnet have numerous dowels and steel plates, with an average value of 40 dowels and 4 steel plates per connection. Therefore, the experimental results obtained from the test setup in Figure 3 with the highest amount of dowels were considered more representative and deemed more appropriate as input for the FE modeling in the present study. These experimental results show that the converging values for the stiffness per shear plane per dowel are significantly lower than the predictions by Equation (1). The test setup in Figure 3b was subjected to cyclic tensile loading, cyclic compressive loading as well as fully reversed loading. The experimental results obtained from the different connections for these loading conditions are characterized by variability. However, based on the test results, a stiffness value per shear plane per dowel of 4 kN/mm is deemed a good approximation.

The rotational stiffness of the beam-to-column connections, $K_{\theta }$, is given by the analytical formula presented in Equation (2), where $n_{dowels}$ is the number of dowels in the connection, $r_{i}$ is the distance from the *i*-th dowel to the center of rotation of the connection, $r_{i}=sqrt\left(x_{i}^{2}+y_{i}^{2}\right)$. With an average beam-column dowel connection configuration of 3 (rows) by 4 (columns), the value of rotational stiffness becomes $K_{\theta }=$ 5818 kNm/rad.

(2) $$K_{\theta }=K_{ser}\sum_{i=1}^{n_{dowels}}r_{i}^{2}=9.3*78200*8\left(shear\;planes\right)*10^{-6}=5818\;\mathrm{kNm}/\mathrm{rad}$$

Frette et al. [20] conducted the energy dissipation tests on the dowelled glulam connections to estimate the damping ratio. The damping ratio results for the setup in Figure 3b were characterized by variability [20,22]. The energy dissipation of connections was calculated from the hysteresis loops obtained by cyclic testing [26]. Based on the experiments, the damping ratios varied in the range 5–12% with an approximate average value of 8%: $\xi_{conn}$ = 5–12% ($\xi_{conn,mean}≃8\%$).

### 3.3. Field Tests

**Figure 4:**
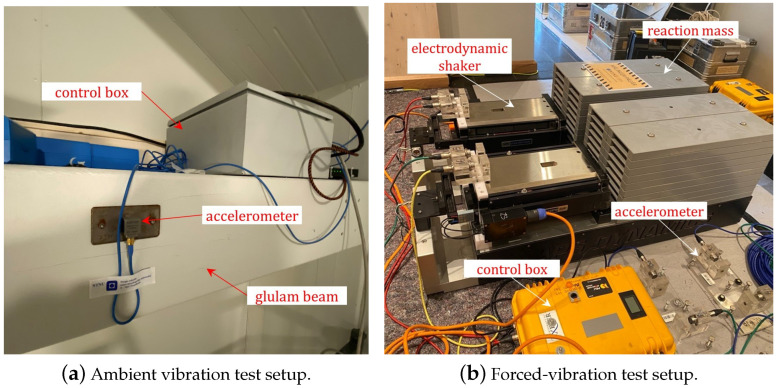
Ambient and forced-vibration tests in the case study building [5].

An extensive full-scale field vibration testing campaign was conducted on Mjøstårnet [5,10]. Two types of vibration tests were included: ambient vibration tests (AVTs, Figure 4a) and forced-vibration tests (FVTs, Figure 4b). The AVT setup included a set of triaxial accelerometers installed in the building, and the acceleration data were continuously collected using the data logger. The forced-vibration tests were conducted using the electrodynamic shakers with a reaction mass of 1 tonne. The results show that the damping ratio is not constant but varies in the range of 0.5–2.0%. The experimental campaign and the methodology for data analysis are described in the authors’ previous study [5], and only the summary of the results is presented here and is shown in Table 1.

### 3.4. Finite Element Modeling

**Figure 5:**
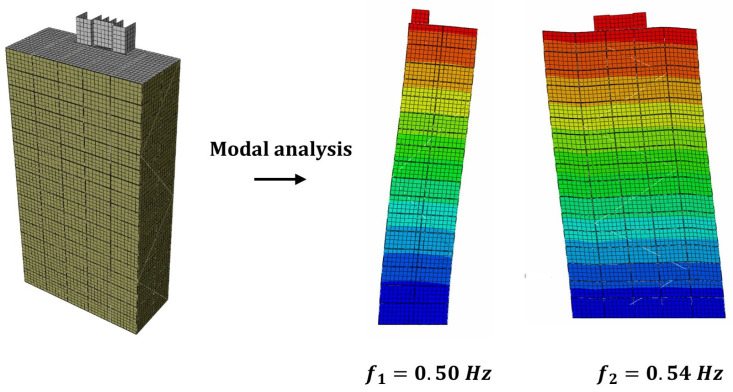
FE Model of Mjøstårnet and the corresponding modal analysis results.

A finite element model of the Mjøstårnet was developed in Abaqus CAE [27]. The glulam elements (beams, columns, and diagonals) were modeled with 1-D linear Timoshenko beam elements. CLT panels, floors, and non-structural partitions were modeled with 2-D linear shell elements. Glulam elements, CLT panels, and prefabricated timber decks were modeled with orthotopic material properties, while reinforced concrete decks and non-structural partitions were modeled with isotropic material properties. The boundary conditions were assigned as pinned supports. The mass properties of the building were estimated using the best engineering judgment, and the material properties of the glulam, CLT, and floor materials were taken from the producer. Figure 5 shows the finite element model of the building and the corresponding modal analysis results. As can be seen from the figure, the mode shapes for the two fundamental modes are translational modes in weak and strong directions, respectively, and the corresponding eigenfrequencies are 0.5 Hz and 0.54 Hz. A mesh sensitivity analysis showed that the accuracy of the result does not improve significantly for a mesh size of 1 meter and below for frame and shell members. More detailed information about the geometry, connection modeling approach, and material properties can be found in the previous study by Tulebekova et al. [10].

#### 3.4.1. Glulam Connection Modeling

**Figure 6:**
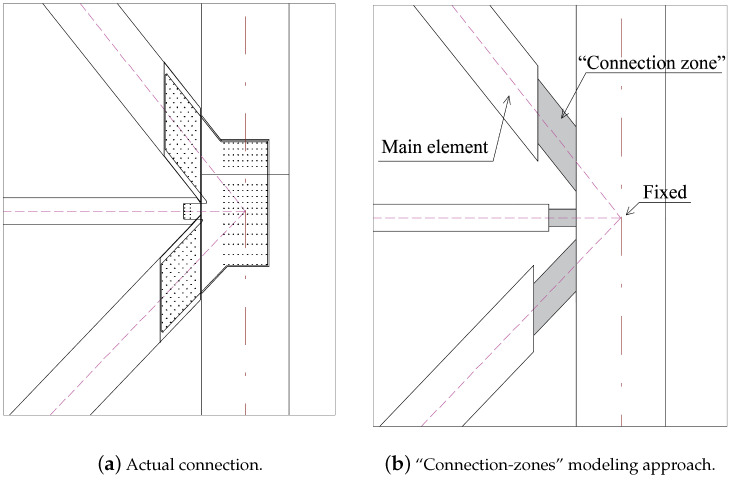
Comparison of actual vs. modeled dowelled connection with slotted-in steel plates [10].

Connections in glulam-frame buildings have a significant impact on the overall dynamic behavior of the structure. It is important to include them in the modeling process, but such detailed models require substantial computational effort and are prone to errors due to intricate geometric details in connections [8,11]. Therefore, making a simplified model of the connections is common, such as assigning generalized elements or springs. In this case study, glulam connections were modeled with “connection-zones”: generalized beam elements with stiffness properties directly dependent on the connected main beam or diagonal elements. Figure 6 shows the actual dowel connection with slotted-in steel plates (Figure 6a) and the corresponding modeling approach with “connection-zones” (Figure 6b). The “connection-zone” elements have arbitrary cross-sectional area and moment of inertia, which allows assigning stiffness properties without specifying the geometry of the section. The stiffness of the connection is dependent on the main connected element through the stiffness reduction factor. This factor allows accounting for reduced rotational and axial stiffness in the “connection zone” and is equal to $I_{red}/I_{main}$ or $A_{red}/A_{main}$, where ${*}_{red}$ and ${*}_{main}$ stand for the “connection-zone” stiffness and main element stiffness, respectively, where the maximum value of the reduction factor is 1.0 and indicates the rigid connection. This approach significantly simplifies the modeling of the actual slotted-in dowelled connection. Still, it preserves the interaction between the glulam elements and the connections, omitted in the traditional pinned connection modeling approach. Further information on the “connection-zone” modeling approach can be found in [10]. The relationship between the stiffness magnitude and the “connection zone” is calculated as shown in Equations (3) and (4).

(3) $$K_{a,diag}=\frac{E_{0}\cdot A_{red}}{L_{red}}$$

(4) $$K_{rot,beam}=\frac{6\cdot E_{0}\cdot I_{red}}{{L_{red}}^{2}}$$

where $K_{a,diag}$ is the axial stiffness of the diagonal connection, $K_{rot,beam}$ is the rotational stiffness of the beam connection, $L_{red}$ is the length of the “connection-zone” element, which is set to be the same as the height of the connected main beam or diagonal element, $A_{red}$ is the cross-sectional area of the diagonal “connection zone”, $I_{red}$ is the moment of inertia of the beam “connection zone”.

#### 3.4.2. Damping Modeling

According to EN1991-1-4 [12], total damping in the structure can be estimated as a sum of structural, material, and aerodynamic damping. In this study, the primary focus is on structural behavior rather than aerodynamic interaction. Therefore, aerodynamic damping is omitted. Thus, only damping in the connections and the main structural elements is considered.

As mentioned earlier, damping in the building is a complex property, which is difficult to model. Therefore, it is common to use Rayleigh damping Equation (5), which provides a simplified approach and is often used by structural engineers. In Equation (5), ξ is the damping ratio, $\omega_{n}$ is the natural angular frequency, α and β are Rayleigh damping coefficients. Rayleigh damping consists of a linear combination of mass- and stiffness-proportional damping matrices and is fairly straightforward to incorporate into a finite element model of the structure [26].

(5) $$\xi =\frac{1}{2}\left(\alpha \omega_{n}+\frac{\beta }{\omega_{n}}\right)$$

The previous experimental and numerical study on composite timber decks [17] has shown that stiffness-dependent Rayleigh damping provides the best estimations of the actual damping properties, whereas mass-dependent Rayleigh damping underestimated the actual values. Additionally, the stiffness-dependent damping can be easily incorporated into the model (see [18]). Therefore, the stiffness-dependent Rayleigh damping model was chosen for this study.

The damping properties in the FE model for glulam timber, connections, and timber decks were defined using the corresponding Rayleigh coefficients in the material property section of the software.

## 4. Parametric FE Study

**Figure 7:**
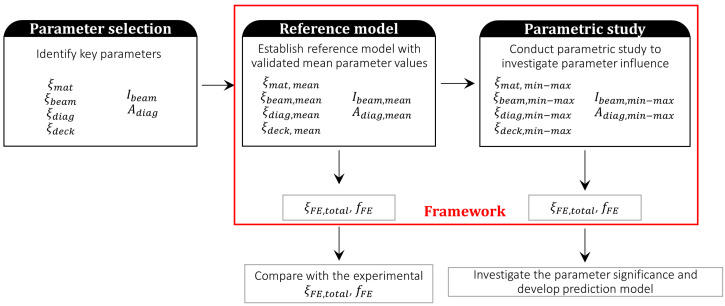
Parametric study stages.

The parametric study is an effective computation technique to analyze the performance of the model and to study the influence of the input parameters on the target properties of the system. The proposed framework for damping estimation is applied to the presented case study to evaluate the performance of the framework and analyze the influence of the input parameters on the global damping ratio of the building. The parametric study was conducted in several stages, as shown in Figure 7. First, the key parameters that can influence the damping ratio of the case study model are identified, and the corresponding mean values and the ranges for the parameters from the previous laboratory experiments or the Eurocode predictions are outlined. Then, the framework is evaluated through application to the reference model. The reference model is the FE model of the building with the mean properties of the selected parameters, which also serves as the statistically representative benchmark for parametric analysis. The resulting damping ratio and fundamental frequency are then compared to the experimental results. In the last stage, the parametric study is conducted by varying the selected parameters within the prescribed range using the One-factor-at-a-time method to identify the most influential parameters on the output damping ratio and develop the prediction model for the case study. A more detailed description is presented in the following sections.

### 4.1. Parameter Selection

The damping ratio of the building is influenced by dynamic properties, such as damping, mass, and stiffness. Since the stiffness-dependent Rayleigh damping model was used in the proposed framework, only stiffness- and damping-related parameters were chosen for the framework evaluation and parametric study. Selected damping ratio parameters include glulam-timber material damping ratio ($\xi_{mat}$), damping ratio in beam and diagonal connections ($\xi_{beam}$ and $\xi_{diag}$), and composite timber deck damping ratio ($\xi_{deck}$). The axial stiffness of the diagonal connections ($A_{diag}$) and rotational stiffness of the beam connections ($I_{beam}$) were shown to be significant stiffness parameters for the global natural frequencies [10], and thus, were selected for the parametric study.

### 4.2. Reference Model

As mentioned earlier, the reference model is the model with mean parameter properties. In this study, the uniform distribution was assumed for all parameters. Thus, the mean value was taken as a midpoint between the limits of the range. Table 2 and Table 3 show the mean values for stiffness and damping parameters (reference model) as well as the ranges for the parameters. Since the connections in this study were modeled with “connection-zones”, it was necessary to convert the stiffness value to the reduction factor using the expressions in Equations (3) and (4). In Table 2, the entire range between 0.2 and 100.0% for connection stiffness parameters was investigated in the parametric study due to large variability in the connections.

The framework was applied to the reference model to compare the resulting fundamental frequency and damping ratio to the experimental results. The resulting fundamental frequency was 0.45 Hz, which is slightly lower than the fundamental frequency of 0.5 Hz from the field experiments. The resulting damping ratio was 1.8%, which is within the range of the field experiment results (0.5–2.0%). Thus, the application of the framework to the case study of Mjøstårnet resulted in realistic global damping and frequency values.

### 4.3. Parametric Study

The parametric study was conducted using the One-factor-at-a-time (OFAT) method. In this method, all parameters remained constant and equal to the reference model values (Table 2 and Table 3), while one parameter was varied within the prescribed range. Additionally, the damping ratio of 0% was added to the parametric study to isolate the other damping parameters.

A parametric study with the OFAT method was automated and performed as follows: the main script was written in Python, v.3.11.5 where the first step was to modify the corresponding parameters in the input Excel file, the second step was to implement the framework (run the Abaqus batch script as a subprocess, output the free decay data from the results of the analysis, and calculate the fundamental frequency and damping ratio using the logarithmic decrement) and finally, record the output frequency and damping back to the Excel file.

## 5. Results and Discussion

**Figure 8:**
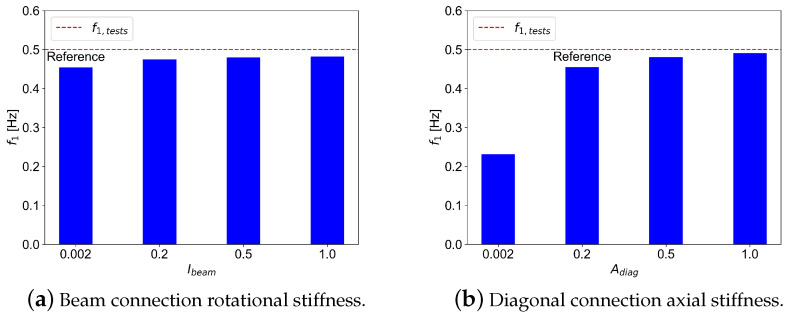
Effect of variation of connection stiffness on the fundamental frequency.

This section presents the results of the parametric analysis. The effect of connection stiffness on the fundamental natural frequency was investigated first. Figure 8 shows the impact of connection stiffness variation on the fundamental frequency. As expected, the axial stiffness of diagonal connections significantly affects the fundamental frequency (Figure 8b), while the rotational stiffness of the beam connections has a negligible effect (Figure 8a).

**Figure 9:**
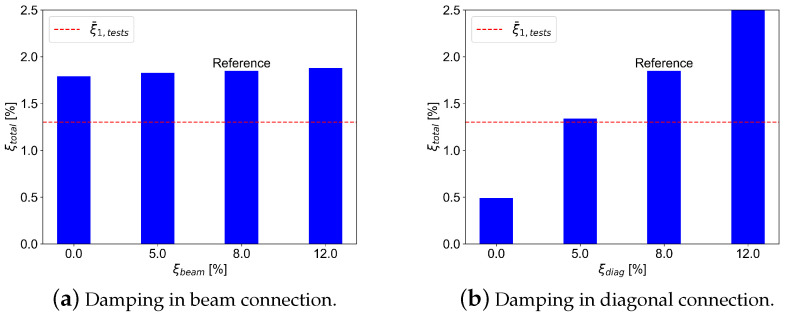
Effect of variation of connection damping on the total damping.

The impact of variation in connection damping on the global damping is shown in Figure 9. Similar to the fundamental frequency, the global damping variation is closely connected to the damping variation in diagonal connections. The variation of the beam connection damping has no significant effect on the global damping. The experimental global damping in the building is not constant but varies and tends to be dependent on the magnitude of excitation. Based on the previously conducted forced-vibration experiments [5], the variation of global damping was found to be in the range between 0.5 and 2.0% with a mean value of approximately 1.3%.

**Figure 10:**
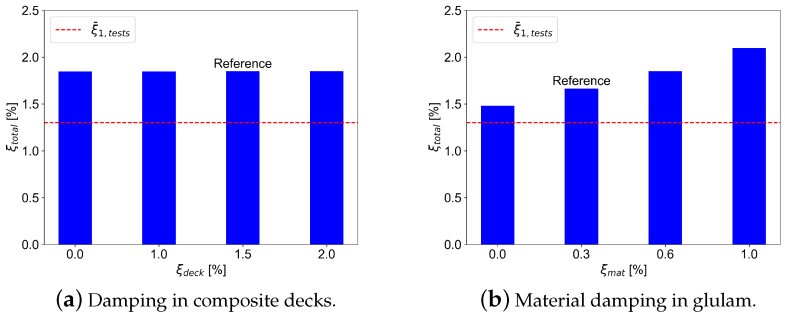
Effect of variation of damping in composite decks (**a**) and glulam-timber material damping (**b**) on the total damping.

The impact of damping in composite timber decks was investigated as well. Figure 10a shows the variation of damping in composite timber decks and the corresponding global damping. As can be seen, damping in timber decks has no impact on the global damping of the building. Figure 10b shows the parametric results for material damping in glulam timber. From observation, a clear dependence of global damping on the glulam material damping can be observed.

**Figure 11:**
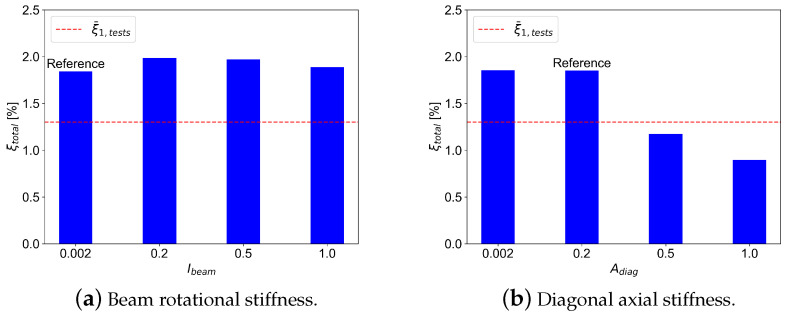
Effect of variation connection stiffness on the total damping.

Finally, the comparison between the variation in connection stiffness and the global damping is presented in Figure 11. A decrease in global damping can be observed as the diagonal stiffness increases, while no significant change can be observed related to the beam rotational stiffness. This tendency may be interpreted as follows: higher stiffness in diagonal connections contributes to higher global stiffness, which then leads to less energy dissipation and less damping ratio.

Based on the parametric study of the effect of damping in the components of a glulam-frame building on global damping, it can be observed that damping in the diagonal connections as well as the material damping in the glulam timber are the two most important contributors to the total damping. Moreover, the total damping increases linearly with an increase in both diagonal connection damping and glulam-timber material damping. Linear regression analysis was used to determine the relationship between the two damping components and the global damping. Equation (6) shows the final expression for the total damping in terms of the material damping and the axial connection damping. This expression is specific to the case study of the tall glulam-frame building.

(6) $$\xi_{total}=0.180\cdot \xi_{diag}+0.702\cdot \xi_{mat}$$

**Figure 12:**
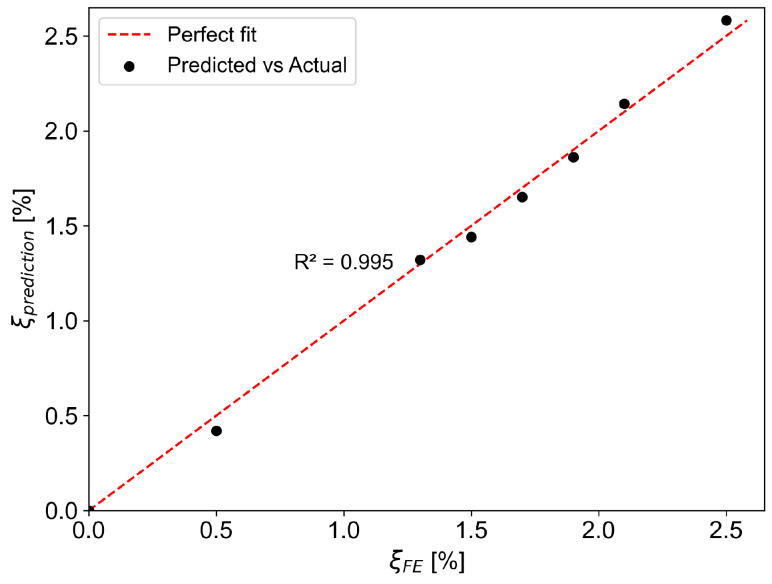
Prediction of damping vs. the FE damping.

Figure 12 shows the goodness of fit with the coefficient of determination value close to 1.0 ($R^{2}=1.0$). The computed coefficients with the corresponding confidence intervals are 0.702 ± 0.113 for the $\xi_{mat}$ coefficient and 0.180 ± 0.008 for the $\xi_{diag}$ coefficient. The confidence interval for the $\xi_{diag}$ coefficient is quite narrow, indicating a higher degree of precision of the estimation. On the other hand, the confidence interval for the $\xi_{mat}$ coefficient is significantly larger, suggesting a higher degree of uncertainty concerning the contribution to the global damping. Nevertheless, the range of variation is still acceptable for engineering applications where the real-life structure is subject to natural variability. According to Equation (6), when using the means values, $\xi_{diag}$ = 8% and $\xi_{mat}$ = 0.6%, the predicted value and the corresponding confidence interval of the total damping ratio in the case study becomes $\xi_{total}$ = 1.86% ± 0.12%.

## 6. Conclusions

This study presented the application of a framework for estimating the damping ratio in glulam-frame buildings and its application in the Mjøstårnet building as a case study. The damping in the building was modeled using stiffness-dependent Rayleigh damping, and the stiffness of the connections was modeled using the “connection-zone” approach. The stiffness and damping values were obtained from the results of small- and large-scale experimental measurements, which have been performed previously. The damping in diagonal connections and the material damping in glulam were found to be the governing parameters in the parametric analysis. The obtained damping ratio using the proposed framework was within the range of the experimental results from ambient and forced-vibration measurements in situ. Based on parametric analysis results, a linear expression for the prediction of the global damping ratio in the Mjøstårnet was obtained. The use of the framework in the presented case study demonstrated that a proper damping ratio estimation can be achieved using a linear-elastic finite element model and stiffness-dependent Rayleigh damping.

The proposed methodology assumes linear model behavior, which, while straightforward in implementation for design purposes, may not fully capture the actual behavior of the building. In real life, buildings can exhibit nonlinear behavior due to material properties, nonlinearity in connections (e.g., an initial slip for small force in connections with oversized holes), and amplitude dependencies. For example, in the previous study by the authors, amplitude-dependent damping was observed from full-scale vibration tests [14]. Potential damage can also affect the stiffness and damping properties. Moreover, timber is inherently hygroscopic material, and moisture can impact the performance and dimensions of timber members and the response of connections. While the building envelope usually isolates the timber members and connections from external environmental changes, improper internal ventilation or leakages may cause increased moisture content and lead to swelling or biological degradation of timber. The study does not take into account the fatigue due to static loading (creep) or repeated loading, which is deemed more relevant in timber bridges. Future work should incorporate the nonlinear damping behavior in the building model when subjected to various wind loading conditions, as well as introduce damage models to account for wind-induced accumulated damage in the building and investigate the impact of moisture content on the connection stiffness and properties.

## Figures and Tables

**Table 1:** Summary of field vibration test results [5].

	Frequency [Hz]		Damping Ratio [%] *	
**Mode**	**AVT**	**FVT**	**AVT**	**FVT**
1	0.50	0.50	1.10–1.35	0.5–2.0
2	0.54	0.53	1.66–2.00	0.5–3.0
3	0.82	0.82	1.00–1.35	-

* The variation in the AVT and FVT damping ratio was due to amplitude-dependence of damping. See study by [5].

**Table 2:** Stiffness properties in the Reference model and corresponding ranges for the parametric study.

Parameter	Experiment/EC	Connection Zone	Range
$I_{beam}$	2900 kNm/rad	0.2%	0.2–100%
$A_{diag}$	1280 kN/mm	20%	0.2–100%

**Table 3:** Damping properties in the Reference model and corresponding ranges for the parametric study.

Parameter	Reference Value	Range
$\xi_{mat}$	0.6%	0.3–1.0%
$\xi_{beam}$	8%	5–12.0%
$\xi_{diag}$	8%	5–12.0%
$\xi_{deck}$	1.5%	1.0–2.0%

## Data Availability

The original contributions presented in this study are included in the article. Further inquiries can be directed to the corresponding authors.
